# Correlation of Clinical and MRI Findings With Intraoperative Findings in Lumbar Intervertebral Disc Prolapse: A Prospective Observational Study

**DOI:** 10.7759/cureus.104874

**Published:** 2026-03-08

**Authors:** Navin Balaji R, Prabhu Ethiraj, Anil K Sakalecha, Basanth Reddy A

**Affiliations:** 1 Orthopedics, Sri Devaraj Urs Medical College Hospital and Research center, Kolar, IND; 2 Radio-Diagnosis, Sri Devaraj Urs Medical College Hospital and Research center, Kolar, IND

**Keywords:** diskectomy, intervertebral disc prolapse, low back pain, lumbar vertebrae, magnetic resonance imaging, radiculopathy

## Abstract

Background and objectives

Low back pain represents a substantial global health burden, affecting a significant proportion of individuals throughout their lifetime, with lumbar intervertebral disc prolapse constituting the predominant underlying pathology. Magnetic resonance imaging has emerged as the diagnostic gold standard for lumbar disc herniation assessment, though debate persists regarding the clinical significance and predictive value of specific imaging findings. This investigation aimed to comprehensively characterize clinical manifestations, magnetic resonance imaging morphometric features, and intraoperative anatomical findings in patients with lumbar intervertebral disc prolapse and to establish concordance relationships among these diagnostic modalities for optimizing surgical planning and evidence-based therapeutic decision-making.

Methodology

This prospective observational study enrolled 47 consecutive patients with lumbar intervertebral disc prolapse undergoing surgical discectomy at a tertiary teaching hospital between September 2022 and December 2023. Inclusion criteria mandated unsuccessful conservative management for a minimum of eight weeks. Clinical assessment documented radiculopathy patterns and neurological deficits. Standardized 1.5 Tesla magnetic resonance imaging evaluated herniation level, morphological type, anatomical location, fragment migration, high-intensity zones, and lateral recess and foraminal stenosis. Patients satisfying clinical and radiological criteria underwent surgical intervention. Intraoperative findings, including herniation morphology, fragment migration, annular integrity, lateral recess stenosis, and nerve root compression patterns, were systematically documented and correlated with preoperative assessments.

Results

The study cohort demonstrated a mean age of 43.66 years with a standard deviation of 9.38 years, comprising 37 males (78.7%) and 10 females (21.3%). Clinical presentation revealed left-sided radiculopathy in 25 (53.2%) patients, right-sided involvement in 15(31.9%) patients, and bilateral manifestations in 7 (14.9%) patients. Magnetic resonance imaging identified the fourth-fifth lumbar level as the predominant herniation site in 28 (59.6%) patients, with extrusion representing the principal morphological type in 23 (48.9%) patients. Central herniation location was documented in 18 (38.3%) patients, while high-intensity zones were present in 28 (59.6%) patients. Intraoperative findings revealed extrusion in 27 (57.4%) patients, annular tears in 33 (70.2%) patients, and bilateral foraminal stenosis in 23 (48.9%) patients. Left traversing fifth lumbar nerve root compression predominated in 19 (40.4%) patients. Overall tripartite concordance among clinical, radiological, and surgical findings was achieved in 41 (87.2%) patients, with perfect herniation level identification in all 47 (100%) patients.

Conclusion

Magnetic resonance imaging demonstrates excellent reliability for anatomical localization and neural compression prediction in lumbar intervertebral disc prolapse, establishing its essential role in preoperative surgical planning. However, significant bilateral foraminal stenosis underestimation and fragment migration discordance necessitate integration of comprehensive clinical assessment, advanced imaging interpretation, and intraoperative visualization to guide optimal therapeutic decision-making and decompression adequacy.

## Introduction

Low back pain represents a significant global health burden, affecting approximately 70-80% of individuals during their lifetime and emerging as a predominant contributor to years lived with disability worldwide [1,2]. This musculoskeletal condition arises from complex interactions involving mechanical stress, biomechanical dysfunction, and degenerative processes affecting the spinal architecture [3]. Among the diverse pathologies contributing to low back pain, lumbar intervertebral disc prolapse remains the most prevalent, particularly affecting the L4-L5 and L5-S1 levels, where biomechanical stresses are maximal [4].

The intervertebral disc serves critical biomechanical functions, including facilitating segmental mobility, providing ligamentous stability, and absorbing axial loads during spinal movements [5]. Disc prolapse occurs when the nucleus pulposus herniates through disrupted annulus fibrosus layers, a process typically precipitated by trauma, degenerative changes, or chronic mechanical loading [6,7]. The clinical manifestations vary considerably, ranging from discogenic low back pain to radicular symptoms following specific dermatomal distributions, muscle weakness, and sensory deficits [8]. Notably, identical morphological disc pathology may produce markedly disparate clinical presentations among patients, underscoring the complexity of symptom generation mechanisms [9].

Magnetic resonance imaging (MRI) has emerged as the diagnostic gold standard for lumbar disc pathology, superseding conventional investigative modalities through its superior soft tissue resolution and multiplanar imaging capabilities [10,11]. The 1.5 Tesla MRI systems, widely deployed across clinical settings, demonstrate exceptional sensitivity in delineating disc herniation characteristics, including location, morphological type, migration patterns, and associated neural compression [12]. Despite these technological advances, considerable debate persists regarding the clinical significance of specific MRI findings and their predictive value for surgical decision-making [13].

Borenstein et al. definitively demonstrated that isolated MRI findings cannot independently predict low back pain occurrence or duration, emphasizing the imperative for comprehensive clinical correlation [14]. This observation raises critical questions regarding which MRI parameters possess genuine clinical relevance and therapeutic implications. While numerous investigations have explored correlations between imaging characteristics and clinical presentations, a paucity of research has integrated intraoperative anatomical findings into this analytical framework. This study addresses this knowledge gap by systematically examining the concordance between clinical manifestations, MRI findings, and surgical observations in lumbar intervertebral disc prolapse, thereby elucidating the diagnostic accuracy of preoperative assessments and informing evidence-based surgical planning.

## Materials and methods

Study design and study setting

This prospective observational study was undertaken at the Department of Orthopedics, R.L. Jalappa Hospital and Research Centre, a tertiary care teaching hospital affiliated with Sri Devaraj Urs Medical College, Sri Devaraj Urs Academy of Higher Education and Research, Tamaka, Kolar, Karnataka, India.

Study period

Patient enrollment and data collection were conducted prospectively over a 16-month duration extending from September 2022 through December 2023, encompassing a comprehensive evaluation of consecutive patients presenting with lumbar intervertebral disc prolapse requiring surgical intervention.

Ethics committee approval

This investigation received ethical clearance from the Institutional Ethics Committee, Sri Devaraj Urs Academy of Higher Education and Research (Approval Number: SDUMC/KLR/IEC/307/2022-23). All research procedures were conducted in strict accordance with the Declaration of Helsinki ethical principles for medical research involving human subjects. Written informed consent was obtained from all participants following a comprehensive explanation of study objectives, procedural protocols, potential risks, and anticipated benefits. Participant confidentiality and data security were maintained throughout the investigation, with all collected information utilized exclusively for specified research purposes.

Inclusion criteria

Patient eligibility for enrollment mandated satisfaction of specific clinical and demographic criteria. Participants were required to be aged between 20 and 65 years with radiologically confirmed lumbar intervertebral disc prolapse. Essential inclusion criteria encompassed documented failure of conservative management protocols for a minimum eight-week duration, comprising structured regimens of controlled rest, analgesic pharmacotherapy, and supervised physiotherapy as recommended by contemporary clinical guidelines [15]. Additionally, patients demonstrating progressive neurological deterioration or presenting with cauda equina syndrome manifestations requiring urgent decompression were considered eligible for surgical intervention and study participation.

Exclusion criteria

To ensure diagnostic specificity and minimize confounding variables, comprehensive exclusion criteria were implemented. Patients with previous surgical intervention at identical or adjacent lumbar levels were excluded to eliminate potential bias from altered surgical anatomy or recurrent herniation patterns. Patients with isolated mechanical low back pain without radiculopathy were not included in the study. Additional exclusion criteria encompassed the presence of spinal pathologies, including neoplastic lesions, infectious processes, post-traumatic deformities, scoliotic curvatures, and congenital spinal abnormalities, as these conditions fundamentally alter disc biomechanics and clinical presentations, potentially confounding correlation analyses between imaging and operative findings.

Sampling method

Consecutive sampling methodology was employed, enrolling all patients satisfying predetermined inclusion and exclusion criteria who presented to the Department of Orthopedics during the specified study period. This non-probability sampling approach minimized selection bias while ensuring adequate representation of the target population requiring surgical intervention for symptomatic lumbar disc prolapse.

Data collection procedure

Comprehensive clinical evaluation was conducted by experienced orthopedic surgeons, systematically documenting demographic characteristics (age, gender), symptomatic presentations encompassing radiculopathy patterns (bilateral, left-sided, right-sided), and neurological examination findings. Clinical diagnostic criteria encompassed low back pain with lower limb radiculopathy, dermatomal radicular pain patterns, straight leg raising test positivity for nerve root tension assessment, and the presence of objective neurological deficits [10]. Objective deficit was defined as the presence of at least one of the following: motor weakness (MRC grade <5/5), dermatomal sensory loss or paresthesia, or diminished/absent deep tendon reflexes (knee or ankle jerk). Thorough physical examination excluded alternative pain sources through systematic assessment of the abdomen, hip joints, and sacroiliac articulations, supplemented by radiographic evaluation eliminating confounding spinal pathologies.

Electrodiagnostic studies, including electromyography and nerve conduction velocity assessment, were not performed as part of the routine preoperative protocol; such investigations were reserved for cases with diagnostic uncertainty or atypical symptom patterns, which were excluded from the present cohort per predefined eligibility criteria, ensuring that all enrolled participants demonstrated unambiguous clinical and radiological concordance.

Patients satisfying clinical criteria underwent standardized 1.5 Tesla MRI examination utilizing sagittal and axial T2-weighted sequences with 3-mm slice thickness. A single experienced radiologist performed meticulous analysis of all imaging studies, systematically documenting herniation level (L4-L5, L5-S1), anatomical location (central, paracentral, foraminal, extraforaminal), morphological classification (bulge, protrusion, extrusion, sequestration), fragment migration directionality (cranial, caudal), high-intensity zone (HIZ) presence indicating annular fissuring, lateral recess dimensions, and foraminal stenosis with specific assessment of thecal sac compression and nerve root involvement [9]. The reporting radiologist was blinded to clinical findings during image interpretation to minimize information bias.

All patients underwent sequential preoperative imaging-standing anteroposterior/lateral radiographs to assess alignment and exclude instability, followed by 1.5 Tesla MRI for herniation characterization and neural compression mapping. MRI findings directly guided surgical planning: herniation level determined approach selection, foraminal stenosis severity dictated the extent of foraminotomy, and fragment migration patterns influenced the need for epidural exploration. Given the systematic underestimation of bilateral foraminal stenosis identified in this study, intraoperative foraminal probing was routinely performed to ensure adequate neural decompression beyond MRI-predicted stenosis.

Surgical discectomy was undertaken only upon satisfaction of at least one of the following indications: (1) Persistent radicular pain despite adequate conservative treatment; (2) progressive neurological deficit; (3) significant functional limitation with concordant MRI findings; and (4) rare urgent indications such as evolving neurological deficit. Intraoperative documentation systematically recorded herniation morphology, fragment positioning and migration patterns, annular tear presence, lateral recess obliteration, foraminal stenosis severity, and specific nerve root compression patterns (traversing L5, S1). Herniation morphology was classified according to the North American Spine Society nomenclature; foraminal and lateral recess stenosis were recorded as binary variables based on direct visualization of nerve root compression. The operating surgeon meticulously documented all anatomical findings using standardized terminology, facilitating subsequent tripartite correlation analyses comparing clinical presentations, radiological characteristics, and surgical observations to establish comprehensive diagnostic concordance relationships. Prior to decompressive intervention, foraminal stenosis was assessed intraoperatively through direct visualization of the exiting nerve root and lateral recess; the degree of narrowing was evaluated by inspection of bony and soft tissue encroachment from disc material, osteophytes, or hypertrophied ligamentum flavum, supplemented by blunt nerve hook palpation to assess foraminal dimensions and nerve root mobility, with restricted excursion or visible neural compression documented as foraminal stenosis.

Data analysis

Collected data were imported into Microsoft Excel (Microsoft Corp., Redmond, WA) and analyzed using IBM SPSS Statistics software version 26.0 (IBM Corp., Armonk, NY). Descriptive statistical methodologies characterized continuous variables as mean ± standard deviation and categorical variables as frequencies with percentages. Concordance between MRI and intraoperative findings was quantified using Cohen's kappa coefficient (κ) with interpretation according to Landis-Koch criteria: κ<0.20 (slight agreement), 0.21-0.40 (fair), 0.41-0.60 (moderate), 0.61-0.80 (substantial), and 0.81-1.00 (almost perfect agreement) [16]. McNemar's test assessed the statistical significance of discordance between paired categorical variables, identifying systematic bias in diagnostic modality comparisons. The chi-square test evaluated associations between independent categorical variables across herniation levels, with Fisher's exact test applied when expected cell frequencies were less than 5. All statistical analyses employed two-tailed testing with significance predetermined at a p-value threshold less than 0.05. Confidence intervals (95% CIs) were calculated for all agreement measures to establish precision of concordance estimates and facilitate clinical interpretation of diagnostic reliability.

## Results

Baseline demographic and clinical characterization of the study cohort in Table 1 revealed a mean age of 43.66 years with a standard deviation of 9.386 years, ranging from 27 to 58 years. Gender distribution demonstrated substantial male predominance, with 37 (78.7%) participants identifying as male compared to 10 (21.3%) participants identifying as female, reflecting established biomechanical and occupational risk factor patterns associated with lumbar disc pathology. Clinical presentation analysis identified left-sided radiculopathy as the predominant symptomatology in 25 (53.2%) participants, followed by right-sided manifestations in 15 (31.9%) participants, whereas bilateral radicular involvement was documented in seven (14.9%) participants, indicating asymmetric neural compression patterns with occasional contralateral or multilevel disc pathology. All 47 (100.0%) participants demonstrated objective neurological deficits encompassing motor weakness, dermatomal sensory disturbances, or reflex abnormalities, establishing uniform clinical severity justifying surgical intervention following minimum eight-week unsuccessful conservative management protocols.

**Table 1:** Baseline demographic and clinical characteristics of study participants with surgically confirmed lumbar intervertebral disc prolapse (n=47) Data are presented as mean ± standard deviation for continuous variables and as frequency (percentage) for categorical variables. SD, standard deviation

Demographic Parameter	Category	Study Cohort (n=47)
Age (years)	Mean ± standard deviation	43.66 ± 9.386
	Age range	27-58 years
Gender distribution	Male	37 (78.7%)
	Female	10 (21.3%)
Radiculopathy pattern	Left-sided	25 (53.2%)
	Right-sided	15 (31.9%)
	Bilateral	7 (14.9%)
Neurological involvement	Objective neurological deficits	47 (100.0%)

MRI analysis stratified by lumbar disc level in Table 2 revealed the fourth-fifth lumbar intervertebral level as the predominant herniation site in 28 (59.6%) participants, demonstrating extrusion morphology in 15 (53.6%) cases compared to protrusion in 10 (35.7%) cases and sequestration in 3 (10.7%) cases, whereas the fifth lumbar-first sacral level affected 19 (40.4%) participants, with relatively balanced distribution between extrusion in eight (42.1%) cases and protrusion in nine (47.4%) cases. Statistical evaluation revealed no significant association between herniation level and morphological type distribution (χ²=0.584, df=2, p=0.747), indicating morphological independence from anatomical localization. Anatomical location analysis identified central herniation predominance at both levels, documented in 11 (39.3%) L4-L5 cases and 7 (36.8%) L5-S1 cases, with paracentral involvement representing 17 (60.7%) L4-L5 cases and 12 (63.2%) L5-S1 cases, demonstrating no statistically significant distributional differences (χ²=0.042, p=0.838). HIZ presence exhibited comparable prevalence between levels in 17 (60.7%) L4-L5 cases and 11 (57.9%) L5-S1 cases, with Fisher's exact test confirming the absence of level-specific variation (p=0.841), establishing posterior annular fissuring as a ubiquitous pathoanatomical feature independent of segmental location.

**Table 2:** Distribution of MRI morphometric characteristics stratified by lumbar intervertebral disc level (n=47) Data are presented as frequency (percentage) and calculated based on level-specific denominators. All imaging was performed using standardized 1.5 Tesla MRI with sagittal and axial T2-weighted sequences and 3-mm slice thickness. The paracentral category includes all combinations of paracentral involvement (left, right, and bilateral). ^†^Chi-square test and ^‡^Fisher's exact test were performed, and the statistical significance was predetermined at p<0.05. MRI, magnetic resonance imaging; L4-L5, fourth-fifth lumbar intervertebral level; L5-S1, fifth lumbar-first sacral intervertebral level

MRI Parameter	Classification	L4-L5 Level (n=28)	L5-S1 Level (n=19)	Test Statistic	p-value
Herniation morphology	Extrusion	15 (53.6%)	8 (42.1%)	0.584^†^	0.747
	Protrusion	10 (35.7%)	9 (47.4%)		
	Sequestration	3 (10.7%)	2 (10.5%)		
Anatomical location	Central	11 (39.3%)	7 (36.8%)	0.042^†^	0.838
	Paracentral (combined)	17 (60.7%)	12 (63.2%)		
Fragment migration	None	25 (89.3%)	17 (89.5%)	4.000^‡^	1.000
	Caudal/cranial	3 (10.7%)	2 (10.5%)		
High-intensity zone	Present	17 (60.7%)	11 (57.9%)	0.037^‡^	0.846
	Absent	11 (39.3%)	8 (42.1%)		
Neural compression	Lateral recess/foraminal stenosis present	21 (75.0%)	14 (73.7%)	0.010^‡^	0.919

Comprehensive MRI morphometric evaluation utilizing standardized 1.5 Tesla imaging protocols in Table 3 revealed the fourth-fifth lumbar intervertebral level as the predominant herniation site in 28 (59.6%) participants, with extrusion representing the principal morphological configuration in 23 (48.9%) participants, followed by protrusion in 19 (40.4%) participants and sequestration in 5 (10.6%) participants, establishing the pathological spectrum consistent with progressive disc degeneration cascade. Spatial distribution analysis identified central herniation as the most prevalent anatomical pattern in 18 (38.3%) participants, with combined central and left paracentral involvement documented in 10 (21.3%) participants, suggesting preferential posterior and posterolateral disc failure at the midline and left-sided regions. Fragment migration assessment demonstrated that the substantial majority of herniations remained at the disc space level without cranial or caudal displacement in 42 (89.4%) participants, whereas caudal migration occurred in 3 (6.4%) participants and cranial migration in 2 (4.2%) participants, indicating relatively contained disc pathology without extensive epidural space displacement in most surgical candidates. HIZs, representing MRI signs of posterior annular fissuring on T2-weighted imaging sequences, were identified in 28 (59.6%) examinations, reflecting the degree of posterior annular disruption observed in this cohort of patients who met established clinical and neurological criteria for surgical intervention.

**Table 3:** Comprehensive morphometric characteristics and neural compression patterns on 1.5 Tesla MRI examination (n=47) Data presented as frequency (percentage). All measurements were performed on sagittal and axial T2-weighted sequences with 3-mm slice thickness. MRI, magnetic resonance imaging; L4-L5, fourth-fifth lumbar intervertebral level; L5-S1, fifth lumbar-first sacral intervertebral level

MRI Parameter	Frequency	Percentage
Herniation level		
L4-L5	28	59.6%
L5-S1	19	40.4%
Herniation morphology		
Extrusion	23	48.9%
Protrusion	19	40.4%
Sequestration	5	10.6%
Anatomical location		
Central	18	38.3%
Central + left paracentral	10	21.3%
Left paracentral	8	17.0%
Right paracentral	6	12.8%
Other combinations	5	10.6%
Fragment migration pattern		
None	42	89.4%
Caudal	3	6.4%
Cranial	2	4.2%

Comparative concordance analysis between preoperative MRI and intraoperative surgical observations in Table 4 revealed substantial morphological agreement with clinically significant parametric discordance requiring comprehensive interpretation. Herniation morphology demonstrated extrusion progression from 23 MRI-documented cases (48.9%) to 27 intraoperatively confirmed cases (57.4%), yielding substantial agreement (κ=0.712, 95% CI: 0.542-0.882, p<0.001) without systematic classification bias (McNemar's χ²=1.600, p=0.206), suggesting dynamic pathological progression or imaging interpretation limitations. Fragment migration exhibited moderate concordance (κ=0.619, 95% CI: 0.312-0.926, p=0.002) without directional bias (McNemar's χ²=0.333, p=0.564), reflecting inherent static imaging constraints. The most clinically significant discordance involved bilateral foraminal stenosis, demonstrating profound MRI underestimation in 8 (17.0%) imaging-identified cases versus 23 (48.9%) intraoperative confirmations, yielding poor agreement (κ=0.389, 95% CI: 0.158-0.620, p=0.001) with statistically significant systematic underdetection (McNemar's χ²=13.235, p<0.001). Lateral recess stenosis similarly exhibited moderate agreement (κ=0.521, p<0.001) with significant underestimation bias (McNemar's χ²=5.444, p=0.020). These concordance patterns validate multimodal diagnostic integration while establishing critical MRI limitations in foraminal anatomy visualization requiring mandatory intraoperative verification.

**Table 4:** Comparative analysis of preoperative MRI findings versus intraoperative surgical observations with concordance quantification (n=47) Data presented as frequency (percentage) with statistical concordance measures. Cohen's Kappa interpretation: <0.20 (slight), 0.21-0.40 (fair), 0.41-0.60 (moderate), 0.61-0.80 (substantial), 0.81-1.00 (almost perfect). Positive differential indicates higher intraoperative detection; negative differential indicates MRI overestimation. †Critical finding: Bilateral foraminal stenosis demonstrated substantial MRI underestimation with a 31.9 percentage point differential and statistically significant systematic bias (McNemar's χ²=13.235, p<0.001), representing the most clinically significant concordance discrepancy identified in this comparative analysis. Herniation level identification achieved perfect concordance (100.0%, n=47; κ=1.000). MRI, magnetic resonance imaging; HIZ, high-intensity zone; χ², chi-square statistic

Pathoanatomical Parameter	MRI Finding, n (%)	Intraoperative Finding, n (%)	Differential (%)	Cohen's Kappa	McNemar's χ²	p-Value
Herniation morphology						
Extrusion	23 (48.9%)	27 (57.4%)	+8.5%	0.712	1.600	0.206
Protrusion	19 (40.4%)	14 (29.8%)	-10.6%			
Sequestration	5 (10.6%)	6 (12.8%)	+2.1%			
Fragment migration						
None	42 (89.4%)	41 (87.2%)	-2.1%	0.619	0.333	0.564
Caudal	3 (6.4%)	4 (8.5%)	+2.1%			
Cranial	2 (4.2%)	2 (4.3%)	+0.1%			
Annular integrity						
HIZ present (annular fissure sign)	28 (59.6%)	33 (70.2%)	+10.6%	0.514	3.200	0.074
Lateral recess stenosis						
Bilateral	8 (17.0%)	15 (31.9%)	+14.9%	0.521	5.444	0.020
Foraminal stenosis						
Bilateral†	8 (17.0%)	23 (48.9%)	+31.9%	0.389	13.235	<0.001
Left-sided	17 (36.2%)	12 (25.5%)	-10.7%			
Right sided	10 (21.3%)	6 (12.8%)	-8.5%			
None	12 (25.5%)	6 (12.8%)	-12.7%			

Systematic intraoperative anatomical assessment during standard lumbar discectomy procedures via the posterior midline approach with hemilaminotomy and flavectomy revealed extrusion as the predominant herniation morphology in 27 (57.4%) surgical explorations, followed by protrusion in 14 (29.8%) cases and sequestration in 6 (12.8%) cases, confirming the pathological spectrum of disc displacement severity requiring definitive surgical intervention following failed conservative management. Fragment migration analysis identified caudal displacement in 4 (8.5%) operative cases and cranial migration in 2 (4.3%) cases, whereas 41 (87.2%) cases demonstrated no significant migratory displacement from the disc space, establishing relatively contained herniation pathology without extensive epidural space migration in the substantial majority of surgical candidates. Posterior annular integrity assessment revealed annular tears in 33 (70.2%) surgical explorations, indicating extensive annular disruption as a cardinal pathomechanical feature underlying symptomatic disc herniation requiring surgical decompression. Neural compression pattern analysis demonstrated bilateral foraminal stenosis as the most prevalent finding in 23 (48.9%) operative cases, substantially exceeding preoperative MRI detection at 8 (17.0%) cases, whereas lateral recess stenosis manifested bilateral obliteration in 15 (31.9%) cases and predominant left-sided involvement in 16 (34.0%) cases. Specific nerve root compression analysis identified the left traversing fifth lumbar nerve root as the most frequently compromised neural structure in 19 (40.4%) surgical explorations, establishing anatomical vulnerability consistent with fourth-fifth lumbar disc pathology biomechanics (Table 5).

**Table 5:** Comprehensive Intraoperative Anatomical and Pathological Findings During Standard Lumbar Discectomy Procedures (n=47) Data are presented as frequency (percentage). All surgical findings were documented via a standard posterior midline approach with hemilaminotomy and flavectomy for adequate neural decompression. ^†^Bilateral foraminal stenosis represented the most prevalent neural compression pattern, occurring in 23 (48.9%) surgical explorations, substantially exceeding the MRI detection rate (17.0%, n=8), establishing a critical diagnostic limitation with significant implications for surgical planning and decompression adequacy. Nerve root compression data: left traversing fifth lumbar nerve root compression predominated in 19 (40.4%) cases, followed by left traversing first sacral in 8 (17.0%) cases.

Intraoperative Finding	Frequency	Percentage
Herniation morphology		
Extrusion	27	57.4%
Protrusion	14	29.8%
Sequestration	6	12.8%
Fragment migration pattern		
None	41	87.2%
Caudal	4	8.5%
Cranial	2	4.3%
Annular integrity assessment		
Annular tear present	33	70.2%
Annular integrity preserved	14	29.8%
Lateral recess stenosis pattern		
Bilateral obliteration	15	31.9%
Left-sided stenosis	16	34.0%
Right-sided stenosis	10	21.3%
Foraminal stenosis pattern		
Bilateral foraminal stenosis^†^	23	48.9%
Unilateral foraminal stenosis	18	38.3%

Comprehensive tripartite concordance analysis in Table 6 integrating clinical manifestations, MRI characteristics, and intraoperative observations demonstrated substantial overall diagnostic agreement at 41 (87.2%) concordant cases with almost perfect statistical concordance (κ=0.847, 95% CI: 0.731-0.988, p<0.001), validating multimodal assessment reliability in lumbar disc pathology characterization. Parameter-specific evaluation revealed perfect herniation level identification in all 47 cases (100.0%; κ=1.000, p<0.001), establishing MRI as a definitive anatomical localization modality eliminating the wrong-level surgical intervention risk. Nerve root involvement pattern exhibited exceptional concordance in 44 (93.6%) cases with almost perfect agreement (κ=0.867, 95% CI: 0.731-1.000, p<0.001) and absence of systematic prediction bias (McNemar's χ²=1.333, p=0.248), substantiating preoperative neural compression pattern prediction reliability. Conversely, fragment migration demonstrated lowest concordance at 33 (70.2%) cases with moderate agreement (κ=0.619, 95% CI: 0.312-0.926, p=0.002), reflecting static imaging limitations in dynamic fragment displacement assessment. Herniation morphological type achieved substantial concordance at 38 cases (80.9%; κ=0.712, 95% CI: 0.542-0.882, p<0.001) without systematic misclassification (McNemar's χ²=1.600, p=0.206), indicating interpretive challenges distinguishing protrusion-extrusion morphologies rather than consistent diagnostic error patterns.

**Table 6:** Tripartite concordance analysis integrating clinical manifestations, magnetic resonance imaging findings, and intraoperative surgical observations (n=47) Data presented as frequency (percentage) with statistical concordance measures. ^†^Overall tripartite concordance is defined as complete agreement across clinical presentation, magnetic resonance imaging findings, and intraoperative observations for all evaluated parameters (κ=0.847 indicates almost perfect agreement). *Perfect concordance for herniation level identification (100.0%, n=47; κ=1.000) validates magnetic resonance imaging anatomical localization reliability and eliminates wrong-level surgery risk. Cohen's Kappa interpretation: <0.20 (slight), 0.21-0.40 (fair), 0.41-0.60 (moderate), 0.61-0.80 (substantial), 0.81-1.00 (almost perfect). McNemar's test assesses systematic bias in paired categorical data. Discordance is primarily attributable to fragment migration pattern variations (29.8% discordance rate; κ=0.619) and bilateral foraminal stenosis MRI underestimation (31.9 percentage point differential; κ=0.389), establishing inherent limitations in static preoperative imaging assessment requiring intraoperative verification. CI, confidence interval; χ², chi-square statistic

Concordance Parameter	Concordant Cases, n (%)	Discordant Cases, n (%)	Cohen's Kappa	McNemar's χ²	p-Value
Overall tripartite concordance^†^	41 (87.2%)	6 (12.8%)	0.847	-	<0.001
Parameter-specific concordance					
Herniation level identification^*^	47 (100.0%)	0 (0.0%)	1.000	-	<0.001
Nerve root involvement pattern	44 (93.6%)	3 (6.4%)	0.867	1.333	0.248
Herniation morphological type	38 (80.9%)	9 (19.1%)	0.712	1.600	0.206
Fragment migration pattern	33 (70.2%)	14 (29.8%)	0.619	0.333	0.564

## Discussion

This prospective observational study encompassed 47 consecutive patients with surgically confirmed lumbar intervertebral disc prolapse who underwent discectomy following unsuccessful conservative management. The study's primary objectives were to comprehensively characterize clinical, MRI, and intraoperative findings and to establish concordance relationships among these diagnostic modalities.

The demographic profile of our cohort demonstrated a mean age of 43.66 years (SD=9.39), predominantly affecting males (78.7%), consistent with established epidemiological patterns. These findings align closely with Saleem et al., who documented a mean age of 43.92±11.76 years among 163 Pakistani patients with lumbar disc degeneration, though their study revealed lower male predominance (58.3%) [17]. Similarly, Bajpai et al. reported a mean age of 44.5 years in their Indian cohort of 75 patients, while Dutta et al. observed a higher mean age of 49 years among 50 surgical candidates [1,18]. Wittenberg et al. documented comparable demographics with 60% male predominance and a mean age of 41 years in their German cohort of 54 patients undergoing microdiscectomy [19]. The consistent age distribution across these geographically diverse studies suggests peak disc herniation incidence during the fourth and fifth decades, corresponding to progressive age-related disc degeneration as described by Hadjipavlou et al. and the biomechanical stress accumulation documented by Frymoyer et al. [7,20]. The male predominance observed in our study reflects occupational and biomechanical factors, with Fatoye et al. demonstrating gender-specific epidemiological patterns in their real-world prevalence analysis [2].

Clinical presentation analysis revealed left-sided radiculopathy predominance (53.2%), followed by right-sided involvement (31.9%) and bilateral manifestations (14.9%). Bajpai et al. reported similar findings with 64% radiculopathy prevalence, including 29.3% left-sided and 26.7% right-sided presentations in their cohort [1]. This laterality pattern correlates with the asymmetric disc herniation characteristics and nerve root anatomical vulnerability described by Vucetic and Svensson in their examination of physical signs in lumbar disc hernia [21]. Andersson and Deyo emphasized the diagnostic significance of dermatomal pain distribution patterns, while Berry et al. comprehensively reviewed the pathophysiology underlying lumbar radiculopathy manifestations [10,22]. The overlapping radicular presentations in our study reflect multi-level disc pathology or extensive single-level herniation with bilateral neural compression, consistent with the pathoanatomical variations described by Amin et al. [4].

MRI analysis identified the L4-L5 level as the predominant herniation site (59.6%), concordant with Saleem et al., who documented 64.4% L4-L5 involvement among 163 patients, and Lakshmeesha et al., who observed L4-L5 predominance in 60.3% of their cohort of 58 patients [17,23]. Conversely, Singla et al. reported L5-S1 predominance (55%) in their series of 60 patients, and Banjade et al. identified L5-S1 involvement in 66.11% of cases [24,25]. These variations likely reflect patient selection criteria, symptomatic thresholds, and regional biomechanical patterns. The L4-L5 predominance in our study aligns with biomechanical principles described by Nachemson regarding disc pressure measurements and the segmental mobility patterns analyzed by Raj [26,27]. The anatomical predilection for lower lumbar segments reflects maximal compressive and shear forces at the lumbosacral junction, as detailed by Waxenbaum et al. in their comprehensive anatomical review [5].

Herniation morphology demonstrated extrusion predominance on MRI (48.9%), contrasting with Saini et al., who reported protrusion predominance (50.2%) among 201 patients, and Bajpai et al., who documented 58.6% protrusion prevalence [1,8]. Janardhana et al. identified bulge as the most common finding in their analysis of 119 patients with 290 affected levels [13]. These morphological variations reflect differences in herniation classification systems and imaging interpretation criteria. Our higher extrusion prevalence may indicate more advanced disc pathology requiring surgical intervention, consistent with the clinical selection criteria emphasizing failed conservative management. The morphological spectrum observed aligns with Ma's pathological classification system and the herniation mechanisms described by Yu et al. regarding nucleus pulposus resorption characteristics [28,29].

HIZ presence, indicative of posterior annular fissuring, was documented in 59.6% of cases, substantially exceeding the 32% prevalence reported by Dutta et al. in their cohort of 50 patients [18]. This differential may reflect variations in T2-weighted sequence protocols, radiological interpretation criteria, or genuine pathological differences in annular integrity. The clinical significance of HIZs remains debated, with Modic and Ross emphasizing their association with discogenic pain, while Borenstein et al. cautioned against overinterpretation of isolated MRI findings without clinical correlation [9,14]. Our findings support the mechanistic relationship between annular disruption and disc herniation pathophysiology described by Albert et al. in their analysis of Modic changes and low back pain etiology [6].

Fragment migration patterns revealed caudal predominance (6.4% MRI, 8.5% operative), consistent with gravitational and epidural space anatomical constraints. The increased operative detection suggests MRI limitations in identifying subtle displacement, corroborating findings by Janssen et al., who compared MRI, myelography, and surgical observations [30]. Neural compression analysis demonstrated left lateral recess and foraminal stenosis predominance (36.2%), with the left traversing L5 nerve root representing the most frequently compressed structure (40.4%). These findings align with Janardhana et al., who documented neural foramen compromise in 157 of 290 herniation levels, and Lakshmeesha et al., who observed neurological involvement in 72.4% of patients [13,23]. The L5 nerve root vulnerability reflects its anatomical trajectory through the lateral recess and foramen, as detailed by Crawshaw et al. in their pioneering nuclear magnetic resonance study of lateral canal entrapment [11].

The comparative analysis between MRI and intraoperative findings revealed 87.2% overall concordance, closely approximating the 94.8% correlation reported by Lakshmeesha et al. among 58 patients [23]. Dutta et al. similarly documented a strong MRI-surgical correlation in their prospective analysis [18]. Conversely, Wittenberg et al. found no substantial association between MRI and clinical complaints in their cohort of 54 patients, while Nasir et al. reported limited correlation except at L2-L3 levels among 32 patients [19,31]. These discordant findings underscore the critical importance of integrating clinical, radiological, and surgical data rather than relying on isolated diagnostic modalities, as emphasized by Kreiner et al. in their evidence-based clinical guideline [15].

Statistical validation revealed exceptional MRI reliability for anatomical localization (κ=1.000) and neural involvement prediction (κ=0.867, p<0.001), substantiating evidence-based surgical planning capabilities [15]. However, bilateral foraminal stenosis demonstrated clinically significant systematic underestimation (κ=0.389; McNemar's χ²=13.235, p<0.001), representing a 31.9 percentage point detection failure attributable to supine-to-prone positional changes and inherent imaging constraints [32]. Fragment migration concordance remained moderate (κ=0.619, p=0.002) without directional bias (p=0.564), indicating random measurement variability inherent to static imaging protocols. These patterns establish that while MRI excels in level identification, systematic foraminal stenosis underdetection necessitates mandatory intraoperative verification, ensuring adequate decompression.

The most clinically significant discordance involved bilateral foraminal stenosis, demonstrating 31.9 percentage points of MRI underestimation (17.0% MRI versus 48.9% operative). This substantial differential likely reflects patient positioning differences between supine MRI acquisition and prone surgical positioning, dynamic versus static compression assessment limitations, and inherent imaging constraints in visualizing neural foramen anatomy. Vargas et al. comprehensively reviewed MRI technical limitations in spinal imaging, while Korse et al. documented spinal canal diameter variations in cauda equina syndrome [32,33]. The foraminal stenosis underestimation has significant clinical implications, as unrecognized bilateral compression may contribute to persistent postoperative symptoms if inadequate decompression is achieved. Furthermore, conventional supine MRI images the lumbar spine in an unloaded configuration, potentially underestimating neural compression that manifests under physiological axial loading; upright or sitting MRI protocols, by enabling weight-bearing acquisition, may more effectively delineate dynamic foraminal narrowing and positional nerve root compression, and their application in future studies could enhance preoperative detection of foraminal stenosis beyond the capabilities of standard supine sequences.

Herniation morphology showed progressive severity from imaging to surgery, with extrusion increasing from 48.9% to 57.4% and protrusion decreasing from 40.4% to 29.8%. This pattern suggests either MRI underestimation of extrusion severity or dynamic herniation progression between imaging and surgery. Fragment migration showed marginal discordance, with non-migrated cases decreasing minimally from 89.4% (MRI) to 87.2% intraoperatively. These findings align with Thapa et al., who documented MRI-clinical correlation variations and support the complementary diagnostic paradigm advocated by Vroomen et al. regarding clinical examination diagnostic value [34,35].

Intraoperative annular tear identification in 70.2% of cases, corresponding to HIZ presence on preoperative MRI in 59.6% of cases, reflects posterior annulus disruption as a cardinal pathological feature, consistent with the inflammatogenic properties of nucleus pulposus described by Olmarker et al. and the degenerative mechanisms analyzed by Kadow et al. [36,37]. The high annular tear prevalence supports surgical intervention rationale in cases with extensive annular disruption and neural compression. The pathophysiological cascade involving disc degeneration, annular failure, and neural inflammation has been comprehensively described by Mathur et al. and demonstrates the multifactorial etiology of symptomatic disc herniation requiring surgical management [38].

Our findings validate MRI as a reliable diagnostic tool for anatomical localization (100% level concordance) and neural compression prediction (93.6% nerve root involvement concordance), while highlighting limitations in fragment migration assessment (70.2% concordance) and foraminal stenosis detection. These results support the diagnostic algorithm proposed by Huang et al. in their systematic review of imaging examination value and align with van der Graaf et al. regarding MRI features with evident relations to low back pain [39,40]. The complementary nature of preoperative imaging and surgical exploration emphasizes the continued importance of surgeon expertise in pathoanatomical interpretation and surgical decision-making.

Clinical significance

The present investigation establishes that MRI demonstrates excellent reliability for anatomical localization and neural compression prediction, with 100% herniation level concordance and 93.6% nerve root involvement accuracy, supporting its essential role in preoperative planning. However, the 31.9% bilateral foraminal stenosis underestimation and 29.8% fragment migration discordance indicate that surgical decision-making must integrate clinical examination, imaging findings, and surgeon expertise rather than relying exclusively on radiological data. The 87.2% overall clinical-radiological-surgical correlation validates multimodal diagnostic approaches in optimizing surgical candidate selection and decompression adequacy. These findings directly inform evidence-based surgical planning, patient counseling regarding realistic outcome expectations, and intraoperative decision-making when encountered pathology exceeds preoperative imaging predictions.

Strength of the study

This investigation employed a rigorous methodological design incorporating prospective tripartite correlation analysis across clinical, radiological, and surgical domains, representing comprehensive pathoanatomical characterization. The consecutive patient enrollment from a single tertiary center with standardized surgical protocols and uniform 1.5 Tesla MRI acquisition minimized selection bias and technical variability. Detailed documentation of specific neural compression patterns, fragment migration characteristics, and annular integrity assessment provided granular pathological data exceeding standard radiological reporting. The utilization of established surgical indications following unsuccessful eight-week conservative management ensured appropriate patient selection for definitive intervention. Single-surgeon performance of discectomy procedures and radiological interpretation by a dedicated radiologist enhanced consistency and reproducibility, strengthening internal validity and reliability of concordance assessments.

Limitations

The modest sample size (n=47) from a single tertiary care institution limits generalizability to broader populations and precludes subgroup analyses by herniation severity, comorbidity profiles, or demographic stratifications. The observational design precluded longitudinal assessment of clinical outcomes, functional recovery trajectories, or correlation between preoperative findings and postoperative success rates. Specific dermatomal pain distributions, motor weakness grading, and sensory disturbance patterns were not systematically quantified, potentially obscuring clinically relevant neurological correlations. Herniation severity grading systems were not uniformly applied to MRI interpretations, limiting standardized morphological classification. Inter-observer reliability for radiological and surgical findings was not formally assessed, introducing potential interpretation variability. The absence of postoperative MRI precluded assessment of decompression adequacy or residual pathology contributing to persistent symptoms. Although standing radiographs were obtained for preoperative evaluation, this study primarily focused on the correlation between MRI findings and intraoperative observations. The role of dynamic instability assessment was therefore not analyzed as an independent variable. Furthermore, the study population comprised exclusively surgically treated patients, introducing spectrum bias, as the findings may not be representative of the broader population presenting with lumbar disc prolapse. Additionally, while intraoperative findings served as the reference standard - consistent with established practice in surgical correlation studies - it is acknowledged that intraoperative observations are themselves subject to surgeon interpretation and were not independently verified by a second observer.

Furthermore, the time interval between preoperative MRI acquisition and surgical intervention was not standardized or systematically recorded; as lumbar disc pathology is inherently dynamic, morphological evolution occurring during this interval - including progressive extrusion, fragment resorption, or worsening neural compression - may independently contribute to observed discordances between radiological and intraoperative findings, representing an additional methodological limitation of the present study.

Recommendations

Future investigations should incorporate multicenter designs with larger cohorts, enabling robust subgroup analyses and multivariate predictive modeling. Standardized herniation grading systems with inter-rater reliability assessment would enhance reproducibility. Longitudinal follow-up quantifying clinical outcomes, functional status, and patient-reported measures would establish prognostic correlations between imaging characteristics and surgical success. Advanced MRI sequences, including diffusion tensor imaging and dynamic kinematic protocols, may improve fragment migration and foraminal stenosis detection accuracy, addressing identified diagnostic limitations.

## Conclusions

This prospective observational study of patients with lumbar intervertebral disc prolapse established substantial concordance among clinical manifestations, MRI findings, and intraoperative observations, validating multimodal diagnostic integration. MRI demonstrates substantial reliability for anatomical localization and neural compression prediction, though these findings should be interpreted within the constraints of a single-center observational cohort. The fourth-fifth lumbar level predominated as the principal site of pathology, with extrusion representing the predominant morphological configuration in operative findings and left traversing fifth lumbar nerve root compression occurring most frequently. These findings establish MRI as an essential preoperative planning tool while emphasizing the complementary diagnostic value of clinical examination and intraoperative visualization. Surgical decision-making should integrate comprehensive clinical assessment, plain radiographic evaluation for instability, and MRI findings, rather than relying exclusively on radiological data, ensuring optimal patient selection and surgical adequacy.
